# Possible Case of Novel Spotted Fever Group Rickettsiosis in Traveler Returning to Japan from India

**DOI:** 10.3201/eid2206.151985

**Published:** 2016-06

**Authors:** Ichiro Takajo, Tsuyoshi Sekizuka, Hiromi Fujita, Ayako Kawano, Takeshi Kawaguchi, Motohiro Matsuda, Kazuyoshi Kubo, Shunichi Miyauchi, Kunihiko Umekita, Yasuhiro Nagatomo, Makoto Kuroda, Tomohiko Takasaki, Akihiko Okayama, Shuji Ando

**Affiliations:** University of Miyazaki, Miyazaki, Japan (I. Takajo, A. Kawano, T. Kawaguchi, M. Matsuda, K. Kubo, S. Miyauchi, K. Umekita, Y. Nagatomo, A. Okayama);; National Institute of Infectious Diseases, Tokyo, Japan (T. Sekizuka, M. Kuroda, T. Takasaki, S. Ando);; Mahara Institute of Medical Acarology, Tokushima, Japan (H. Fujita)

**Keywords:** rickettsia, infectious disease, genome, bacteria, bacterial infection, spotted fever group, SFG, Japan, travel, India, japonica, kellyi, Candidatus, argasii, heilongjiangensis, honei, parkeri, sibirica, africae, conorii, slovaca, indica

## Abstract

A 60-year-old woman experienced fever, headache, rash, and altered vision after returning to Japan from India. Testing detected elevated antibody titers to spotted fever group rickettsia; PCR on blood yielded positive results for the rickettsial outer membrane protein A gene*.* We isolated a unique rickettsial agent and performed a full-genome analysis.

Various types of spotted fever group (SFG) rickettsioses have been reported worldwide (*1*). Common symptoms include fever, headache, intense myalgia, and skin rash (*2*). Even though cases of spotted fever have occurred in Japan, physicians often face difficulty diagnosing the disease in febrile patients because of the unusual or unfamiliar symptoms associated with SFG rickettsia.

In January 2011, a 60-year-old woman from Japan who was undergoing treatment for diabetes mellitus stayed in a suburban area of Bangalore in South India for 1 week. She went camping and did other outdoor activities. She recalled that there were many opportunities to be bitten by insects; however, she could not specify the types of insects to which she may have been exposed. On her first day back in Japan (day 1), she experienced general malaise and loss of appetite. A fever (38°C) and skin rash appeared on day 7. She consulted a local clinic and underwent evaluation. Blood test results indicated thrombocytopenia (91 × 10^9^ thrombocytes/L), liver dysfunction (elevated aspartate aminotransferase [92 U/L] and alanine transaminase [97 U/L]), and elevated C-reactive protein levels (206 mg/L). Splenomegaly was evident on abdominal sonography. On day 12, the woman was admitted to Miyazaki University Hospital (Miyazaki, Japan) with a high fever (39.4°C), headache, altered vision with eye floaters, and rash (Figure 1, panel A). Proteinuria, glycosuria, thrombocytopenia (92 × 10^9^ thrombocytes/L), liver dysfunction (elevated aspartate aminotransferase [95 U/L] and alanine aminotransferase [93 U/L]), and hyponatremia (sodium 127 mmol/L) were observed, and levels of serum procalcitonin (1.9 ng/mL) and C-reactive protein (198 mg/L) were elevated. Her diabetes was poorly controlled (glucose 40.5 mmol/L).

**Figure 1:**
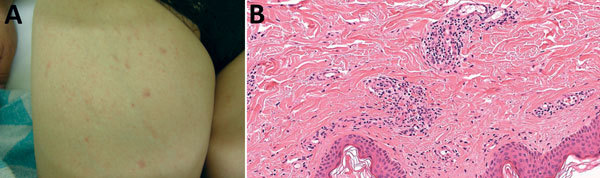
Rash and skin biopsy of a 60-year-old female traveler who had returned to Japan from India, January 2011. A) Rash on the inner side of the right thigh. B) Skin biopsy showing infiltration of inflammatory cells (mainly lymphocytes) around the capillaries, associated with hemorrhagic changes (hematoxylin-eosin staining, original magnification ×400).

A skin biopsy of the rash was performed on day 13; infiltration of inflammatory cells (mainly lymphocytes) around the capillaries, associated with hemorrhagic changes, was observed (Figure 1, panel B). No eschars were detected. Given the patient’s travel history to India and her signs and symptoms, we considered acute infectious diseases such as dengue fever, chikungunya fever, and typhoid fever. Ceftriaxone (2 g/d) and levofloxacin (500 mg/d) were administered. Subsequently, the fever subsided, and platelet count, liver function, and C-reactive protein level returned to normal. However, laboratory findings ruled out the principal tropical infectious diseases. Blood smear specimens tested negative for malaria parasites. Blood culture tested negative for bacteria, thus ruling out typhoid fever. Dengue and chikungunya virus infections were ruled out on the basis of serologic tests and antigen detection. Although the rash, bilateral lower limb edema, and visual alterations with eye floaters persisted, the patient was discharged on day 27.

A serum sample revealed apparent positivity to *Rickettsia conorii* Malish 7 and *R. japonica* YH on day 19 (Table). Therefore, SFG rickettsia was strongly suspected. By day 40, the patient’s rash and edema persisted, and her visual alterations had become more severe. Because persistent SFG rickettsiosis was suspected, additional oral treatment with minocycline (100 mg/d) was administered for 14 days. By day 48, the remaining symptoms resolved.

**Table:** IgG titers to rickettsiae detected by indirect immunofluorescence assay conducted on a serum sample from a 60-year-old female traveler who had returned to Japan from India, January 2011*

Species	Day 13	Day 19	Day 40
*Rickettsia japonica* (YH)	–	1:640	1:640
*R. conorii* (Malish7)	–	1:2,560	1:5,120
*R. typhi* (Wilmington)	–	1:40	1:160
*R. prowazekii* (Brainl)	–	1:40	1:80
*Orientia tsutsugamushi* (Karp)	–	–	–
*O. tsutsugamushi* (Gilliam)	–	–	–


*–, negative.

PCR test results for the acute-phase whole blood sample collected on day 13 were positive for the outer membrane protein A (*ompA*) gene for SFG rickettsia (*3*,*4*). Direct nucleotide sequencing of PCR products (GenBank accession no. LC089865) yielded a profile different from any known *Rickettsia* spp. but identical to that of *Rickettsia* sp. CMCMICRO 1–4 (GenBank accession nos. HM587248.1–HM587251.1) (*5*). We successfully isolated an SFG rickettsial agent, designated as strain Tenjiku01, from acute-phase whole blood (collected on day 13) using the shell vial method with L929 cells. The *ompA* sequence of the isolate was identical to that of the clinical sample (Technical Appendix 1 Figure). In addition to partial sequencing of PCR products (GenBank accession nos.: *16S rRNA,* LC089861; *17K-Da*, LC089862; *geneD*, LC089863; *gltA*, LC089864), we performed whole-genome analysis. The partial sequence of *ompA* from our PCR products was 100% similar to the CMCMICRO *ompA* sequence (*5*) and 98.4% similar to the *Candidatus* R. kellyi *ompA* sequence (*6*). Other genes with high sequence homology were as follows: *gltA* with uncultured *Rickettsia* sp. LIC4275 (99.7% homology, accession no. KT153042); *rrs* with *R. slovaca* 13-B (99.7%, NR_074462); *sca4* (geneD) with *R. slovaca* 13-B (98.4%, CP002428); and 17K-Da gene with *R. honei* RB (99.5%, AF060704). The draft genome sequence (≈1.3 Mb, 32 contigs) was obtained with next-generation sequencing (accession nos. BCMR01000001–BCMR01000032). The outer membrane protein B sequence, which was extracted from the contig by next-generation sequencing, was 96.98% similar to that of *R. slovaca* D-CWPP (accession no. CP003375). Results of pan-genome analysis suggested that 586 core genes were shared among 34 *Rickettsia* spp. genomes, and the gene components of Tenjiku01 were highly similar to those of the SFG group (data not shown). A maximum-likelihood phylogenetic tree of concatenated amino acid sequence alignments of the core genes, constructed by using RAxML software version 8.2.0 (http://sco.h-its.org/exelixis/web/software/raxml), indicated that Tenjiku01 belongs to the SFG group and is closely related to *R. honei* RB (Figure 2). Moreover, blastp matrix analysis (http://blast.ncbi.nlm.nih.gov) of 73 *Rickettsia* spp. indicated that 315 core genes of Tenjiku01 showed 98.21%–98.95% homology to those of *R. japonica*, *R. argasii*, *R. heilongjiangensis*, *R. honei*, *R. parkeri*, *R. sibirica*, *R. africae*, and *R. conorii* (Technical Appendix 2).

**Figure 2:**
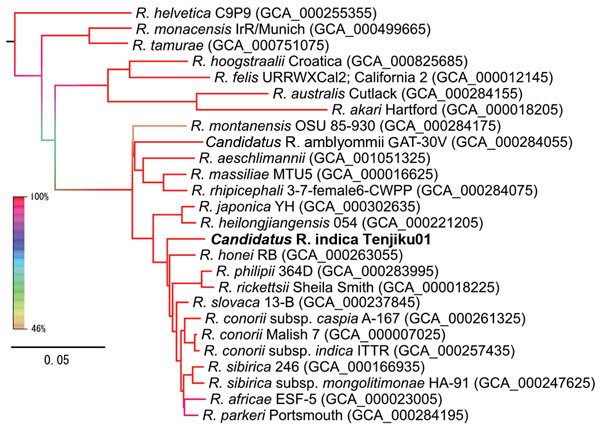
Maximum-likelihood phylogenetic tree of concatenated core genes in 26 *Rickettsia* spp. strains constructed by using RAxML software version 8.2.0 (http://sco.h-its.org/exelixis/web/software/raxml) with 1,000-fold bootstrapping. Boldface indicates isolate from this study. The color of each branch represents the bootstrapping value. GenBank assembly accession numbers are given in parentheses. Scale bar indicates amino acid changes per position.

At first, this patient’s infection partially responded to levofloxacin and ceftriaxone therapy. The efficacy of levofloxacin in treating *Rickettsia* spp. has been described previously (*7*). Therefore, levofloxacin, not ceftriaxone, probably was effective in this patient. After detection of SFG rickettsia antibodies, we performed PCR analysis, which resulted in the final diagnosis; the patient’s remaining symptoms were then successfully treated with minocycline.

Recently, many types of SFG rickettsia, except *R. japonica*, have occurred in Japan (*4*,*8*). An even greater variety of rickettsioses has been reported worldwide (*1*,*9*,*10*), and the incidence of imported rickettsioses has increased in Japan (*11*–*13*).

In our patient, antibody titers to *R. conorii* and *R. japonica* were elevated on day 19, and the titers to *R. conorii* were higher than those against *R. japonica*. Therefore, we suspected that the causative pathogen was closely related to *R. conorii*.

The *ompA* PCR products amplified from clinical samples were identical to the sequences of *Rickettsia* sp. CMCMICRO registered in India. Prakash et al. tested skin biopsy samples for SFG rickettsial genes and concluded that novel species of SFG rickettsia (CMCMICRO1–8) were in their area (*5*). However, isolation of SFG rickettsia has not been reported thus far. Moreover, data on other genes, such as *17KDa*, *gltA*, and *gene D*, are lacking (*5*). Little sequence homology was observed between the *ompA* sequence in our case and that of *Candidatus* R. kellyi (*6*), which is considered to be the most closely related to *Rickettsia* sp. CMCMICRO, according to Fournier’s criteria (*14*). In our case, we successfully isolated SFG rickettsia, *Rickettsia* sp. strain Tenjiku01, from the clinical sample. Comparative genomics suggested that Tenjiku01 could be a novel species because the phylogenetic distance between Tenjiku01 and *R. honei* RB was longer than that between Tenjiku01 and other similar species.

## Conclusions

We successfully diagnosed imported SFG rickettsiosis in a traveler returning to Japan from India on the basis of serology and molecular laboratory techniques. If a patient reports a recent history of travel abroad, physicians should consider SFG rickettsia in the differential diagnosis. Our analysis will help elucidate a variety of rickettsial pathogenicities and biologic characteristics reported worldwide. On the basis of our findings, we propose this isolate as a novel species, *Candidatus* Rickettsia indica.

Technical Appendix 1 Phylogenetic tree of outer membrane protein A sequences in 34 *Rickettsia* spp. strains.

Technical Appendix 2 Results of a blastp matrix analysis of 315 core genes among 73 *Rickettsia* spp. strains.
