# Supplementary material for: Possible Case of Novel Spotted Fever Group Rickettsiosis in Traveler Returning to Japan from India
Source: Emerg Infect Dis. 2016 Jun;22(6):1079–82. doi: 10.3201/eid2206.151985 (PMC4880103; doi:10.3201/eid2206.151985)
Supplement: Technical Appendix 1 — Phylogenetic tree of outer membrane protein A sequences in 34 Rickettsia spp. strains. [file 15-1985-Techapp-s1.pdf]

Article DOI: http://dx.doi.org/10.3201/eid2206.151985

Possible Case of Novel Spotted Fever Group Rickettsiosis in Traveler Returning to Japan from India

Technical Appendix 1

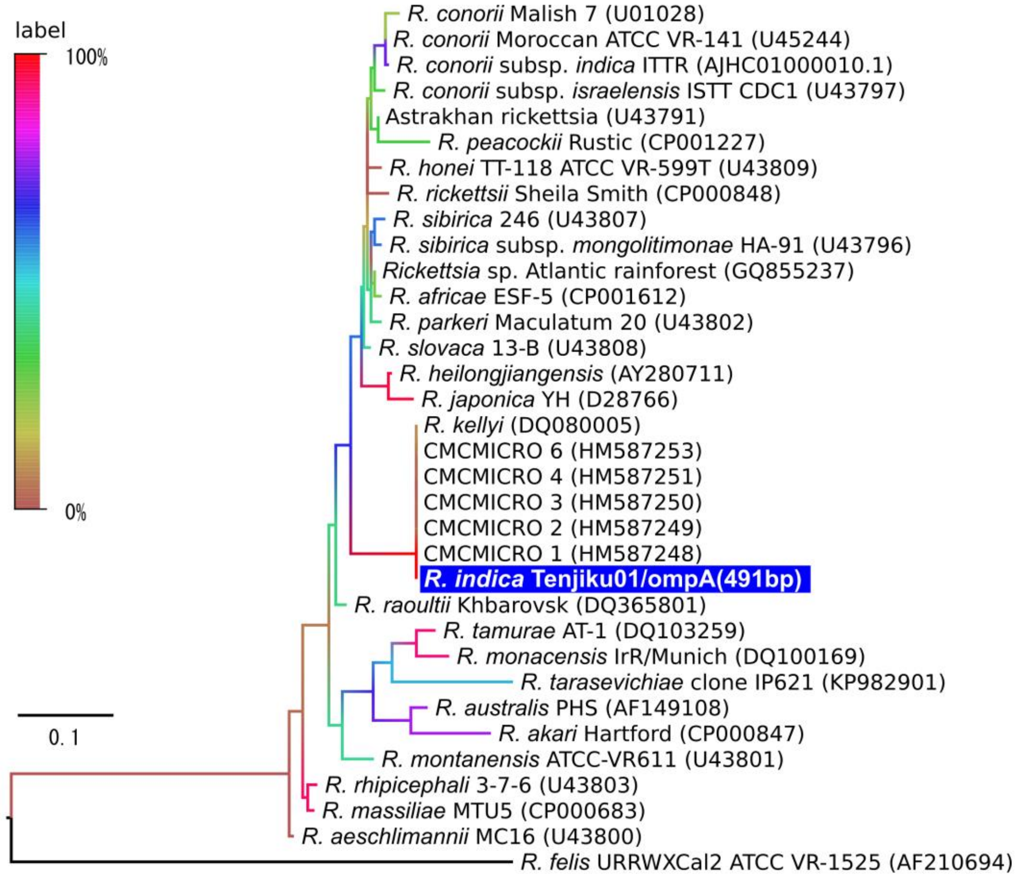

Technical Appendix 1 Figure. Maximum-likelihood phylogenetic tree of outer membrane protein A sequences in 34 *Rickettsia* spp. strains constructed by using MEGA software version 4.0 (http://www.megasoftware.net) with 1,000-fold bootstrapping. The color of each branch represents the bootstrapping value. GenBank assembly accession numbers are given in parentheses. Scale bar indicates the number of nucleotide substitutions per site.
